# Effects of a 12-Week Multidisciplinary Program on Health-Related Physical Fitness and Depressive Symptoms in Overweight and Obese Women Aged Between 45 and 64 Years with Noncommunicable Chronic Diseases

**DOI:** 10.3390/ijerph23060690

**Published:** 2026-05-23

**Authors:** Maria Luiza Amaro Camilo, Enzo Berbery, Endriw Domingues Noronha, Leonardo Vidal Andreato, Luciana Lozza de Moraes Marchiori, Pablo Valdés-Badilla, Braulio Henrique Magnani Branco

**Affiliations:** 1 Graduate Program in Health Promotion, Cesumar University, Maringa 87050-900, Parana, Brazil; malucamilo20@gmail.com (M.L.A.C.); endriwdominguesnoronha@gmail.com (E.D.N.); vidal.leo@hotmail.com (L.V.A.); luciana.marchiori@unicesumar.edu.br (L.L.d.M.M.); 2 Research Group in Health and Education (GESE), Cesumar University, Maringa 87050-900, Parana, Brazil; enzobo14@gmail.com; 3 Interdisciplinary Laboratory of Health Promotion Intervention, Cesumar University, Maringa 87050-900, Parana, Brazil; 4 Cesumar Institute of Science, Technology and Innovation, Cesumar University, Maringa 87050-900, Parana, Brazil; 5 Department of Physical Activity Sciences, Faculty of Education Sciences, Universidad Católica del Maule, Talca 3530000, Chile; valdesbadilla@gmail.com; 6 Sports Coach Career, Faculty of Life Sciences, Universidad Viña del Mar, Viña del Mar 2520000, Chile

**Keywords:** multidisciplinary patient care, health promotion, overnutrition, physical fitness, noncommunicable chronic diseases

## Abstract

**Highlights:**

**Public health relevance: How does this work relate to a public health issue?**

- The growing burden of sarcopenic obesity and noncommunicable diseases in older women critically challenges global healthcare.
- Addressing chronic diseases requires community interventions that integrate physical, nutritional, and psychological strategies.

**Public health significance: Why is this work of significance to public health?**

- A 12-week multidisciplinary intervention (physical exercise, nutritional intervention, and psychoeducation intervention) substantially improved body composition, functional capacity, and depression.
- The intervention increased skeletal muscle mass and reduced visceral fat, preventing frailty, which is often exacerbated by standard weight loss therapies.

**Public health implications: What are the key implications or messages for practitioners, policy makers and/or researchers in public health?**

- Implementing multidisciplinary lifestyle programs offers a scalable and low-cost framework to overcome the growing burden of multimorbidity.
- Psychoeducation must be a core component of weight management to sustain patient adherence to diet and physical exercise.

**Abstract:**

We evaluated the effects of a 12-week multidisciplinary program on health-related physical fitness and depressive symptoms in overweight and obese women (aged 45–64 years) diagnosed with noncommunicable diseases (NCDs). Methods: A longitudinal, pre-experimental, proof-of-concept study was conducted. Thirty-one women completed multidisciplinary interventions [nutritional education or psychoeducation (each once a week), and resistance training (twice a week)]. Body composition (bioelectrical impedance), physical fitness (maximal isometric strength, lower limb strength–endurance, flexibility, and aerobic fitness), and depressive symptoms (PHQ-9) were measured at baseline and post-intervention. Results: Significant improvements in body composition were observed in terms of lean mass (Δ% = 3.7; *p* < 0.001), fat-free mass (Δ% = 3.6; *p* < 0.001), skeletal muscle mass (Δ% = 5.2; *p* < 0.001), fat mass (Δ% = −3.5; *p* < 0.001), body fat percentage (Δ% = −4.7; *p* < 0.001), and visceral fat level (Δ% = −2.9; *p* = 0.012). Physical fitness exhibited a large effect size in the chair stand test (*d* = 0.91) and the 6 min walk test (*d* = 1.22). Depressive symptom scores substantially decreased (*p* < 0.001). Conclusion: The program demonstrated potential efficacy in mitigating sarcopenic obesity, enhancing functional capacity, and reducing depressive symptoms, indicating potential clinical viability for the integrated management of multimorbidity.

## 1. Introduction

Noncommunicable chronic diseases (NCDs) represent a major public health challenge worldwide and accounted for approximately 75% of non-pandemic-related deaths in 2021 [1]. Moreover, rapid population aging has contributed to a higher incidence of conditions associated with progressive loss of functional capacity and an increased risk of mortality, particularly among older individuals [2]. This epidemiological context places increasing pressure on healthcare systems and negatively affects quality of life, especially in middle-income countries with marked social and regional inequalities, such as Brazil [3]. Noncommunicable chronic diseases are characterized by a long duration, multifactorial etiology, gradual onset, and slow progression, and they are often associated with uncertain clinical trajectories [4]. The most prevalent conditions include cardiovascular diseases, systemic arterial hypertension, type 2 diabetes mellitus, obesity, chronic respiratory diseases, and neoplasms [1]. These conditions affect both individual and collective well-being and generate substantial economic costs, particularly in developing countries [5,6].

In 2021, they were estimated to account for at least 43 million deaths globally [1]. Together, these data indicate the need for effective and sustainable public health strategies to address both population aging and the growing burden of chronic conditions [6]. In the context of Brazil, preventive and early interventions may be more economically appropriate than delayed care because they can reduce later treatment demands, prevent hospital use, and preserve limited health system capacity [7,8,9,10]. Evidence from different settings suggests that preventive approaches may reduce later expenditures or provide better value than advanced management does, even when an initial investment is needed [7,8,9]. This rationale is particularly relevant in low-income and middle-income countries, where limited public financing and overburdened hospitals increase the consequences of avoidable late-stage demand [8,10]. In this sense, prevention should be understood as a central public health strategy, rather than a secondary component of care [7,8,9,10].

From this preventive perspective, addressing modifiable risk factors across the life course is especially relevant in Brazil. Aging is accompanied by physiological and behavioral changes, some of which may be attenuated through lifestyle modification. Among these changes, increased body fat, frequently observed with advancing age, is an important risk factor for the development of noncommunicable chronic diseases, and its prevalence varies across the lifespan [11]. Evidence indicates that healthy behaviors, including balanced eating patterns and regular physical activity, are essential for the prevention and management of chronic conditions throughout aging [12,13]. In addition, multidisciplinary interventions that combine nutritional education, physical activity, and other health-promoting actions have shown favorable effects on risk reduction and on the adoption of healthier routines [14,15].

In Brazil, this discussion is particularly important for women, who are more frequently exposed to obesity, multimorbidity, and functional decline during aging, within a context shaped by social, economic, and cultural determinants [16]. Although lifestyle modification is widely recommended in clinical management, the current literature still provides limited information on how different disciplines interact in an integrated manner, particularly among women living with multiple morbidities. Many studies have evaluated nutritional care, physical exercise, and behavioral support separately or in parallel models without full interdisciplinary integration [17]. For this reason, it remains important to develop intervention models that combine these components within a single therapeutic framework. In the present study, this framework is based on the articulation of nutritional reeducation, resistance training, and psychoeducation.

Nutritional reeducation may contribute to metabolic control; resistance training may support physical function; and the management of excess adiposity and psychoeducation may favor self-regulation, coping, and adherence to treatment. Psychoeducational strategies are relevant because they may influence self-efficacy, stress management, and behavioral adherence, in addition to helping to reduce depressive symptoms, which often act as barriers to engagement in exercise and dietary change among individuals with multimorbidity [18,19]. This integrated perspective may be particularly useful in developing countries, where feasible non-pharmacological strategies with potential for application in real-world services are needed. In Brazil, Bolognese et al. [20] reported that group-based nutritional intervention combined with strength exercise improved health-related parameters in middle-aged women, supporting the relevance of integrated lifestyle approaches in this population. Similarly, Marques et al. [21] reported that multidisciplinary interventions were associated with improvements in dietary quality among older Brazilian women.

However, there is still limited evidence in the Brazilian literature regarding interventions that combine nutrition, psychoeducation, and exercise in middle-aged and elderly women with multimorbidity. In this context, the present study contributes by examining the application of a structured multidisciplinary program combining these components in a population with multimorbidity. Therefore, this exploratory proof-of-concept study aimed to evaluate the effects of a 12-week multidisciplinary program integrating nutrition, psychoeducation, and resistance training on health-related physical fitness and depressive symptoms in overweight and obese women aged 45–64 years or older who were diagnosed with noncommunicable chronic diseases. We hypothesized that participation in this integrated program would be associated with improvements in health-related physical fitness and reductions in depressive symptoms in this population.

## 2. Materials and Methods

### 2.1. Experimental Design

A pre-experimental, single-arm, pretest–posttest proof-of-concept study was conducted over a 12-week period. The study was designed as a multidisciplinary intervention program, with assessments performed at baseline (pre-intervention) and immediately after the intervention (post-intervention) in the same group of participants. To ensure methodological transparency and appropriate reporting quality for behavioral and public health interventions, the study was conducted and reported in accordance with the Transparent Reporting of Evaluations with Nonrandomized Designs (TREND) statement [22].

**Figure 1:**
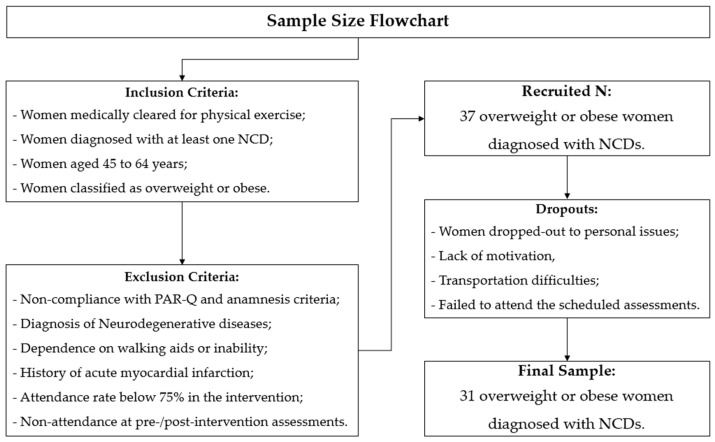
Flowchart of the present study. **Note:** NCDs = noncommunicable chronic diseases.

At the initial meeting, participants were informed about all study procedures and provided written informed consent by signing the informed consent form (ICF). At the same visit, physical activity level was assessed using the International Physical Activity Questionnaire (IPAQ) [23], and depressive symptoms were evaluated using the Patient Health Questionnaire-9 (PHQ-9) [24], as detailed in the following sections. Baseline assessments were conducted during the subsequent week. First, participants underwent an anamnesis performed by a healthcare professional, including medical history collection and blood pressure measurement. They subsequently completed an identification form and underwent anthropometric and body composition assessments, followed by physical fitness tests. The flowchart of the present study is shown in Figure 1.

**Figure 2:**
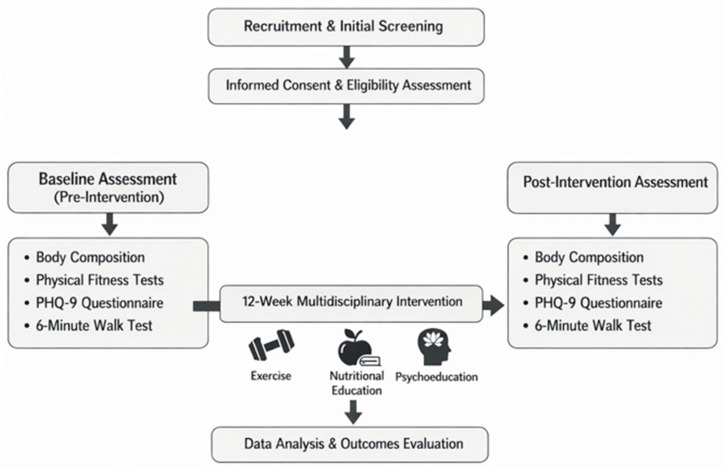
Experimental design/procedures of the study. **Note:** PHQ-9 = Patient Health Questionnaire.

All procedures were performed after medical clearance and under standardized safety conditions. Post-intervention reassessments were conducted after the completion of the 12-week multidisciplinary program, using the same protocols and performed by the same trained evaluators. The measurement reliability was supported by excellent intraclass correlation coefficients (ICCs = 0.96–0.99). The experimental design of the study is shown in Figure 2.

### 2.2. Participants/Sample

Thirty-seven women who were diagnosed with NCDs were initially enrolled in the study. During the intervention, four women withdrew because of personal issues, lack of motivation, or transportation difficulties. Additionally, two women failed to attend the scheduled post-intervention assessments. Consequently, the final analytical sample comprised 31 women who successfully completed and consistently participated in the 12-week program. All participants were enrolled prior to the start of the intervention, and selection was not influenced by baseline performance or anticipated outcomes. As an exploratory proof-of-concept trial, a priori sample size calculation was not performed; however, a post hoc power analysis was conducted to confirm the statistical reliability of the findings (detailed in the Section 2.8 statistical analysis).

The inclusion criteria were women who were medically cleared of physical exercise, were diagnosed with at least one NCD, were aged 45 to 64 years, and were classified as overweight or obese. The exclusion criterion was “yes” to the following items on the Physical Activity Readiness Questionnaire (PAR-Q) [25]: has a physician ever informed you that you have a heart condition and advised you to engage in physical activity only under the supervision of healthcare professionals? Do you experience chest pain during physical activity? Within the past month, have you experienced chest pain during physical activity? Do you experience impaired balance due to dizziness and/or loss of consciousness? Are you aware of any other reason that would prevent you from engaging in physical activity? Diagnosis of neurodegenerative diseases; dependence on walking aids or inability to ambulate independently; history of acute myocardial infarction within the past 12 months; attendance rate below 75% in the intervention sessions; and failure to attend baseline or post-intervention assessments for anthropometric, body composition, physical tests and questionnaires. Table 1 summarizes the characteristics of the study sample at baseline, including descriptive statistics for age, anthropometric measures, body mass index, and the clinical profile of NCDs.

### 2.3. Multidisciplinary Intervention

Participants underwent a 12-week in-person multidisciplinary program (May to July 2024) at the Interdisciplinary Laboratory for Health Promotion Intervention (LIIPS) at UniCesumar. Sessions occurred twice a week (Tuesdays and Thursdays), lasting ~75–90 min each. To ensure a true multidisciplinary approach, the team (nutritionists, psychologists, and physical education professionals) conducted biweekly clinical case meetings to cross-reference participant adherence and integrate dietary, behavioral, and exercise recovery strategies. Participants began each session with either nutritional education or psychoeducation (30 min), followed immediately by resistance training (45–60 min).

### 2.4. Nutritional Intervention

A 12-week, face-to-face, group-based food re-education intervention was conducted, with one session per week lasting 30 min. The intervention was carried out once a week and was interspersed with psychoeducational intervention. The program was developed according to a constructive and progressive framework in which the educational content advanced from basic to more complex topics related to healthy eating behavior. Its theoretical basis was the Dietary Guidelines for the Brazilian Population [26], which emphasize the consumption of fresh and minimally processed foods, the reduction in ultra-processed foods, the promotion of cooking practices and meal planning, and the development of autonomy in food choices.

The intervention was also informed by evidence from two previous studies: one demonstrating that a 12-week multidisciplinary program improved overall diet quality, particularly through increased intake of vegetables and protein-rich foods, and another showing that group nutritional counseling was an effective and feasible strategy for improving dietary intake and health-related outcomes [20,21]. All the sessions were delivered in person by a nutrition professional and followed a standardized format, including a brief review of the previous week, presentation of the central topic, short group discussion, and definition of one practical goal for the following week.

The 12 sessions addressed the following topics in sequence: introduction to food re-education; hunger, satiety, and eating awareness; basic meal structure; food groups and plate composition; meal planning and routine organization; consumption of fruits, vegetables, and protein sources; food classification according to processing level, with emphasis on ultra-processed foods; portion sizes, meal regularity, and diet quality; emotional eating and behavioral triggers; eating outside the home and in social situations; barriers, lapses, and coping strategies; and food autonomy and long-term maintenance [20,21,26]. The intervention emphasized active and participatory educational strategies rather than restrictive dietary prescriptions, aiming to promote food literacy, behavioral change, and the gradual adoption of healthier eating practices.

### 2.5. Psychoeducation Intervention

The behavioral component of the intervention was developed within a cognitive behavioral group therapy (CBGT) framework and was integrated with the nutritional and exercise-related components of the program. The intervention was carried out once a week, alternating with nutritional intervention. The intervention was conducted face-to-face in groups over 12 weeks, with one weekly session lasting 30 min, and was designed to promote adherence to lifestyle modification through the combined use of nutrition education, exercise-related guidance, and psychoeducational strategies. The sessions followed a progressive structure, beginning with a general orientation to the intervention, motivation, and the relationships among eating behavior, physical activity, and emotional health. Subsequent sessions addressed healthy eating principles, meal structure, hunger and satiety awareness, and the role of physical activity in health promotion [27,28].

As the intervention progressed, greater emphasis was placed on the cognitive and behavioral processes underlying lifestyle adherence, including body image, weight stigma, emotional eating, behavioral triggers, stress and anxiety management, and barriers to exercise and dietary compliance. The final sessions focused on behavioral activation, relapse prevention, and the consolidation of autonomous self-regulation strategies aimed at sustaining healthy eating practices and regular physical activity beyond the intervention period. Throughout the program, interactive group discussions were used to enhance self-efficacy, normalize common difficulties, and strengthen participants’ engagement with long-term behavior change [27,28].

### 2.6. Physical Exercise

The supervised exercise protocol consisted primarily of resistance training and lasted 45 to 60 min per session. Training intensity and volume were individually adapted and systematically progressed using the principles of perceived recovery status [29] and gradual progression [30]. Sessions included strength and muscular endurance exercises utilizing free weights and machines, complemented by functional and dual-task neuromotor activities to improve proprioception and coordination. Each session comprised a 6 min general warm-up (light walking), a 45 min main resistance component targeting major muscle groups, and a 6 min cool-down with active stretching. Although predominantly resistance-based, this modality was prescribed because of its potential efficacy in mitigating sarcopenic obesity, improving neuromuscular economy, and enhancing overall gait efficiency, adaptations that translate directly into significant improvements in submaximal aerobic capacity and functional mobility in clinical populations [31].

### 2.7. Health-Related Physical Fitness Tests

#### 2.7.1. Anthropometry and Body Composition

Height and body mass were measured using a stadiometer coupled to a mechanical scale (Welmy^®^, model 104A; Santa Bárbara d’Oeste, São Paulo, Brazil), strictly following the protocols established by Gibson, Wagner and Heyward [32]. Body composition was estimated via a multifrequency, tetrapolar, eight-point tactile bioelectrical impedance analyzer (InBody^®^ Co., model 570; Seoul, Republic of Korea). The following variables were analyzed: lean mass (kg), fat-free mass (kg), skeletal muscle mass (kg), fat mass (kg), body fat (%), and visceral fat level. All measurements were conducted under a controlled laboratory temperature of 24 °C. In cases of menstruation, assessments were specifically rescheduled for the postmenstrual period to guarantee fluid balance and measurement reliability [11,32,33].

#### 2.7.2. Maximal Isometric Handgrip Strength

To assess maximal isometric handgrip strength (MIHS), a digital handgrip dynamometer (Takei Physical Fitness Test^®^, model T.K.K-5101, Tokyo, Japan) was utilized. Each participant was seated with their shoulders in a neutral position, one hand resting on the thigh, and the elbow of the assessed limb fully extended, with the forearm in neutral rotation. The dynamometer’s size and position were individually adjusted. The participants performed three maximal attempts of 3–5 s for each hand, with a 1 min rest interval between attempts. The highest recorded value was utilized for analysis [32,34,35].

#### 2.7.3. Maximal Isometric Lumbar Traction Strength

Maximal isometric lumbar traction strength (MILTS) was evaluated using a traction dynamometer (Takei Physical Fitness Test^®^, Back Strength Dynamometer Type 2, Tokyo, Japan) with a 300 kg capacity. The participants stood on the device platform with their knees slightly flexed, the trunk maintaining a slight forward flexion of approximately 120°, the head aligned with the trunk, and the gaze fixed forward. Hands were positioned at a distance equivalent to the bitrochanteric diameter. Upon the evaluator’s command, participants applied maximal muscular force in the lumbar region for 3–5 s, attempting to pull the support bar (positioned at the patella level) upward to extend the lumbar spine. Three attempts were performed with a 1 min rest interval, and the highest value was recorded [32,34]. The sum of the MILTS of the right and left hands was used for statistical analyses.

#### 2.7.4. Chair Stand Test

Lower limb muscular endurance and functional strength were assessed using the 30 s Chair Stand Test (CST) [36]. Participants started in a seated position with the following posture: the trunk was upright, the feet were flat on the floor positioned shoulder-width apart, and the arms crossed over the chest with the middle fingers placed on the acromia. At the evaluator’s command, participants stood up and sat down as many times as possible within 30 s. Two familiarization attempts were performed prior to the main test.

#### 2.7.5. Sit-And-Reach Test

Lower back and hamstring flexibility was measured using a Wells bench [37]. Participants were seated on a mat with their lower limbs fully extended forward and feet placed parallel to the base of the bench. Participants performed trunk flexion, sliding the centimeter-graduated marker forward with both hands (one placed over the other) while maintaining finger extension and fully extended knees, strictly avoiding any compensatory movements. The test was repeated three times, and the highest value was recorded [32,38].

#### 2.7.6. Six-Min Walk Test (6MWT)

Submaximal aerobic endurance was evaluated using the 6MWT. The test was performed on a 45.7 m course, marked every 4.57 m, in a leveled and ventilated environment equipped with seating, stopwatches, measuring tapes, cones, and staff. Following a detailed explanation, participants walked as fast as possible for six consecutive minutes without interruptions, and the total distance covered was recorded [39]. Heart rate (HR) and peripheral oxygen saturation (SpO_2_) were monitored immediately before and after the test using a portable pulse oximeter (Mediclini^®^, model AS-302-L, Shenzhen, China). Blood pressure (BP) was also strictly monitored before and after the test using a professional digital blood pressure monitor (Omron Healthcare Corporation^®^, model HBP-1120, Dalian, China) [40].

#### 2.7.7. Patient Health Questionnaire-9 (PHQ-9)

Depressive symptomatology was evaluated using the Patient Health Questionnaire-9 (PHQ-9) [25], which has been validated for the Brazilian context [41]. The instrument assesses nine criteria: anhedonia; depressed mood; sleep problems; lack of energy; changes in appetite; feelings of guilt; concentration difficulties; psychomotor retardation or agitation; and suicidal thoughts. Items are rated on a four-point Likert scale, ranging from 0 (‘not at all’) to 3 (‘nearly every day’). The total score was calculated by summing all the items (range: 0–27), where higher scores indicate greater symptom severity.

### 2.8. Statistical Analysis

Data are presented as the absolute frequency (N), relative frequency (%), mean (M), standard deviation (SD), 95% confidence interval (95%CI), median, and interquartile range. Normality was assessed through visual inspection of Q-Q plots and confirmed using the Shapiro-Wilk test. Delta relative values (Δ%) were also calculated [pre minus post-values (%)]. Comparisons between baseline and post-intervention measurements were analyzed using the Wilcoxon signed-rank test (for body composition, PHQ-9 and physiological responses) or the paired Student’s t test (for physical fitness tests), depending on the data distribution. Effect sizes for clinical magnitude were derived using Cohen’s *d* for parametric variables [42] and the equation r = Z/√N for nonparametric outcomes [43]. For the parametric data, d values of approximately 0.20, 0.50, and ≥0.80 were interpreted as small, medium, and large effects, respectively. For nonparametric data, r values of approximately 0.10–0.29, 0.30–0.49, and ≥0.50 were classified as small, medium, and large effects, respectively [42,43]. To definitively address the exploratory nature of the study, a post hoc power analysis was conducted using G*Power software (version 3.1.9.7) [44]. For the final sample size (*n* = 31), an alpha error probability of 0.05, and considering the large effect sizes observed in the primary outcomes (*d* > 0.80), the statistical power (1 − β) exceeded 0.95. Analyses were conducted using complete-case data, with no imputation procedures applied for missing values. All analyses were performed using SPSS version 30.0.0 (IBM Corp^®^, Armonk, NY, USA), with the significance level set at *p* < 0.05.

## 3. Results

**Figure 3:**
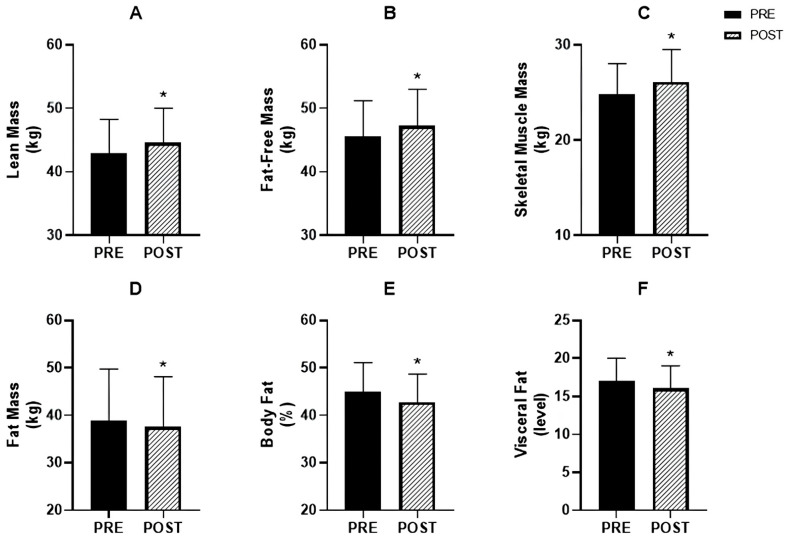
Values of body composition components obtained before and after multidisciplinary intervention in overweight and obese women aged ≥ 45 years with noncommunicable chronic diseases. **Note:** Data are expressed as the mean and standard deviation; Panel (**A**) = lean mass (kg); Panel (**B**) = fat-free mass (kg); Panel (**C**) = skeletal muscle mass (kg); Panel (**D**) = fat mass (kg); Panel (**E**) = body fat (%); Panel (**F**) = visceral fat level; * = *p* < 0.05 = difference between pre- and post-intervention.

The values of body composition components obtained at the pre- and post-multidisciplinary intervention assessments in overweight and obese women aged 45–64 years with NCDs are shown in Figure 3. All the components significantly changed after the intervention: lean mass [r = −0.80 (large effect); Δ% = 3.7; *p* < 0.001]; fat-free mass [r = −0.72 (large effect); Δ% = 3.6; *p* < 0.001]; skeletal muscle mass [r = −0.85 (large effect); Δ% = 5.2; *p* < 0.001]; fat mass [r = −0.63 (large effect); Δ% = −3.5; *p* < 0.001]; body fat percentage [r = −0.74 (large effect); Δ% = −4.7; *p* < 0.001]; and visceral fat level [r = −0.45 (medium effect); Δ% = −2.9; *p* = 0.012].

Table 2 presents the results of health-related physical fitness tests in overweight and obese women aged 45–64 years with NCDs. Compared with the baseline test, all the assessed tests revealed significant changes at the post-intervention assessment: MIHS (*p* = 0.010; *d* = 0.50; medium effect), MILTS (*p* < 0.001; *d* = 0.65; medium effect), CST (*p* < 0.001; *d* = 0.91; large effect), sit-and-reach test (*p* = 0.028; *d* = 0.42; small effect), and 6MWT (*p* < 0.001; *d* = 1.22; large effect).

Table 3 shows the values of the physiological responses, including heart rate, peripheral oxygen saturation, and blood pressure, monitored before and immediately after the 6MWT at the pre- and post-multidisciplinary intervention assessments in overweight and obese women aged 45–64 years with NCDs. In addition, comparisons were performed for the 6MWT variables in the rest condition (before and after 12 weeks of intervention), with the following results: HR [r = −0.05 (small effect); Δ% 0.68; *p* = 0.791]; SpO_2_ [r = −0.12 (small effect); Δ% −0.19; *p* = 0.493]; SBP [r = −0.32 (medium effect); Δ% 9.20; *p* = 0.078]; DBP [r = −0.01 (small effect); Δ% 7.61; *p* = 0.961]; and subsequently the 6MWT before and after 12 weeks of intervention; after this test, HR [r = −0.01 (small effect), Δ% 1.56; *p* = 0.975]; SpO_2_ [r = −0.19 (small effect); Δ% 0.63; *p* = 0.287]; and SBP [r = −0.32 (small effect), Δ% 10.85; *p* = 0.074], or DBP [r = −0.06 (small effect); and Δ% 4.61; *p* = 0.726].

**Figure 4:**
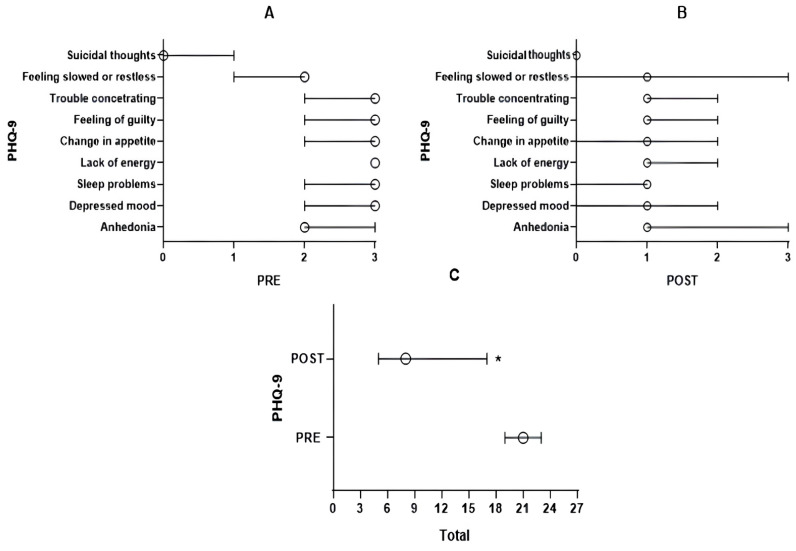
Results of responses to each item and total scores of the Patient Health Questionnaire-9 before and after multidisciplinary intervention in overweight and obese women aged 45–64 years with noncommunicable chronic diseases. **Note:** Data are expressed as median and interquartile ranges; PHQ-9 = Panel (**A**) = PHQ-9 item scores at pre-intervention; Panel (**B**) = PHQ-9 item scores at post-intervention; Panel (**C**) = total PHQ-9 score at pre- and post-intervention; * = *p* < 0.05 = difference between pre- and post-intervention.

The descriptive (individual item scores and total score) and inferential results obtained using the PHQ-9 in overweight and obese women between 45 and 64 years with NCDs at the pre- and post-multidisciplinary intervention assessments are presented in Figure 4. At the post-intervention assessment, women reported significantly lower levels of depressive symptoms than at the pre-intervention assessment did [r = −0.84 (large effect); *p* < 0.001].

## 4. Discussion

The present proof-of-concept study aimed to examine whether a 12-week multidisciplinary intervention integrating nutritional education, psychoeducation, and resistance training was associated with changes in health-related physical fitness and depressive symptoms in overweight and obese women aged 45–64 years or older with noncommunicable chronic diseases. Overall, the findings indicate that participation in the program was accompanied by a consistent pattern of favorable pre–post changes in a pre-experimental design. Specifically, participants showed improvements in body composition, including increases in lean mass, fat-free mass, and skeletal muscle mass, together with reductions in fat mass, body fat percentage, and visceral fat level. In parallel, significant gains were observed across the main domains of physical fitness, including maximal isometric strength, lower-limb functional performance, flexibility, and walking capacity, whereas depressive symptom scores substantially decreased after the intervention. In contrast, the physiological responses measured immediately before and after the 6 min walk remained largely unchanged, suggesting that the most evident short-term adaptations were concentrated in morphological, functional, and psychological outcomes rather than in these acute cardiorespiratory parameters (which may be related to the low volume of cardiorespiratory training). Taken together, these findings are consistent with the study hypothesis, indicating that this integrated multidisciplinary strategy may represent a promising approach for improving physical and mental health indicators in women with multimorbidity, although the results should be interpreted within the exploratory limits of a pre-experimental, single-arm design.

The body composition results indicate that the intervention was associated with a favorable remodeling profile, characterized by significant increases in lean mass, fat-free mass, and skeletal muscle mass, together with significant reductions in fat mass, body fat percentage, and visceral fat level. This pattern is clinically relevant in women with multimorbidity because it suggests that the intervention was associated not only with weight-related changes but also with an improved balance between the metabolically protective and metabolically deleterious compartments. In practical terms, the simultaneous increase in muscle-related variables and reduction in adiposity-related variables is especially important in middle-aged and older women, for whom excess adiposity and progressive functional decline frequently coexist [16]. These findings are consistent with the rationale that integrated lifestyle interventions may produce broader health gains than isolated strategies do [14,15], and they are also aligned with previous studies showing that multidisciplinary or group-based interventions combining nutritional care and exercise can improve health-related outcomes in women with obesity and older adults [20,21]. In addition, the present findings are consistent with recent evidence indicating that resistance-based exercise is an important strategy for improving body composition while helping preserve or increase muscle-related compartments during fat loss interventions in individuals with obesity [44].

Significant improvements were observed in all the health-related physical fitness tests. Maximal isometric handgrip strength, maximal isometric lumbar traction strength, chair stand performance, and sit-and-reach scores improved significantly after the intervention, indicating gains in upper-body strength, trunk/lumbar strength, lower-limb functional performance, and flexibility. These findings are compatible with the central role of resistance training in the intervention protocol and reinforce the functional relevance of this exercise modality in populations exposed to obesity, multimorbidity, and age-related decline [45,46]. From a public health perspective, these changes are meaningful because muscular strength and functional performance are closely related to autonomy, mobility, and the maintenance of independence later in life. It is also plausible that the multidisciplinary structure of the program contributed to these improvements by promoting adherence to exercise and lifestyle change, since psychological and behavioral support may reduce barriers that commonly limit long-term engagement in diet and physical activity programs among individuals with chronic conditions [14,18,19,20,21].

The improvement in the 6 min walk distance deserves specific consideration. Although the intervention focused predominantly on resistance training rather than formal aerobic training, the distance covered in the 6MWT increased significantly after the program. These findings suggest that the intervention was associated with better submaximal functional capacity, possibly through mechanisms related to improved neuromuscular efficiency, lower-limb strength, and movement economy rather than through classic cardiorespiratory conditioning alone [31]. In this sense, the increase observed in the chair stand test may be particularly relevant, since greater lower-limb functional strength may reduce the relative effort required for walking tasks and thereby improve performance during a submaximal field test. This interpretation is consistent with previous evidence showing that resistance-oriented training may positively influence functional mobility and walking-related performance in women with obesity and aging-related conditions [31,44].

In contrast to the significant increase in the 6 min walk distance, the physiological variables associated with the 6MWT, namely, heart rate, peripheral oxygen saturation, systolic blood pressure, and diastolic blood pressure measured before and immediately after the test, did not significantly improve. This absence of statistically significant changes may be explained by the specific characteristics of the exercise intervention. The program was structured primarily around resistance training, with most of the sessions devoted to strength and strength-endurance exercises, while the cardiorespiratory component was limited to brief warm-up and cool-down periods and did not include a structured volume of aerobic training [30,31]. Accordingly, although this intervention model appears to have been sufficient to improve functional walking performance, it may not have provided a strong enough cardiorespiratory stimulus to induce measurable changes in the acute physiological responses assessed around the 6MWT. Thus, the lack of significant differences in these variables should not necessarily be interpreted as a contradiction of the functional findings, but rather as a result consistent with the specificity of the training stimulus applied in this proof-of-concept program.

Depressive symptoms significantly decreased after the intervention, indicating that the program was also associated with meaningful improvement in mental health. These results are particularly relevant because depressive symptoms frequently coexist with obesity, chronic diseases, and functional limitations and may act as important barriers to adherence to exercise and nutritional treatment [18,19,20,21]. In the present study, the reduction in PHQ-9 scores may reflect the combined contribution of regular supervised exercise, group-based psychoeducation, and nutritional guidance delivered within the same therapeutic framework. The psychoeducational component may have been especially important because it addressed motivation, emotional health, body image, behavioral triggers, and long-term self-regulation strategies [27,28]. This interpretation is in line with the literature suggesting that cognitive behavioral and multicomponent group interventions can contribute to improvements in depressive symptoms and behavioral adherence in women with obesity and related chronic conditions [18,19,44]. Taken together, these findings support the view that integrated multidisciplinary approaches may be particularly useful when the goal is to address the interrelated physical and psychological burdens of multimorbidity in aging women.

This study has several limitations that should be acknowledged: (1) Its pre-experimental, single-arm, pretest–posttest design precludes causal inference, as the absence of a concurrent control group does not allow the observed changes to be attributed exclusively to the intervention. Accordingly, threats to internal validity, such as maturation effects, regression to the mean, Hawthorne effects, and uncontrolled concurrent lifestyle changes, cannot be excluded; (2) the use of a small convenience sample composed exclusively of overweight and obese women aged 45–64 years or older with noncommunicable chronic diseases limits generalizability; (3) no long-term follow-up was performed, preventing conclusions regarding the sustainability of the observed benefits. In addition, because the intervention combined nutritional education, psychoeducation, and resistance training in an integrated manner, the specific contribution of each component could not be determined; and (4) another limitation mentioned is dropout, which is related to transportation difficulties and a lack of motivation to participate in scientific study. However, no dropout analysis was performed because of the small number of non-completers, which limits the assessment of potential attrition bias; (5) the lack of control over the consumption of antidepressants or other drugs is also listed as a limitation; and (6) another limitation of the present study was the assessment of body composition using BIA. Although this method is widely applied because of its practicality and non-invasive nature, important constraints must be considered, particularly in obese populations. One major limitation is related to the assumption of constant hydration of fat-free mass. As demonstrated by Kyle et al. [47], variations in fat-free mass hydration frequently observed in individuals with obesity can lead to systematic errors in body composition estimates. Moreover, the predictive equations used in BIA devices are often derived from non-obese populations, which further compromises their validity when applied to individuals with higher adiposity levels. In addition, the accuracy of BIA appears to be influenced by the degree of obesity. Frankenfield et al. [48] reported inconsistencies in body composition estimates when compared to reference methods, particularly as BMI increases. These findings suggest that BIA may be less reliable in individuals with moderate to severe obesity, limiting its applicability in this population. A critical distinction must be acknowledged when comparing BIA to dual-energy X-ray absorptiometry (DEXA), the current gold standard for body composition assessment. Unlike DEXA, which uses low-dose X-ray beams to directly visualize and partition tissue into fat mass, lean mass, and bone, BIA simply measures electrical resistance through body water and subsequently applies predictive formulas, based on age, weight, and height—to estimate fat levels. This fundamental methodological difference has substantial implications for measurement precision. Another relevant issue concerns physiological alterations in body water distribution and external variables affecting measurement reliability. According to Dehghan and Merchant [49], the accuracy of BIA is highly dependent on hydration status, which can be significantly altered in obese individuals because of factors such as inflammation, fluid retention, and changes in extracellular and intracellular water compartments. Furthermore, BIA results can fluctuate significantly based on hydration levels, recent meals, skin temperature, and recent exercise: variables that do not substantially influence DEXA measurements. In contrast, DEXA remains highly consistent regardless of these physiological and environmental factors, contributing to its superior reliability. These deviations from standard assumptions in BIA may substantially reduce the precision of the method, especially in epidemiological settings. The margin of error between these methods is also noteworthy. DEXA is considered the gold standard with a margin of error typically around 1–2%, whereas BIA errors can range from 3 to 5% or higher. Moreover, BIA has been shown to systematically underestimate fat mass in overweight individuals or overestimate it in lean individuals, further limiting its discriminatory capacity across the body composition spectrum. These findings suggest that BIA invites a substantial margin of error, particularly when used as the sole method for body composition assessment in heterogeneous populations. Overall, these limitations highlight the need for cautious interpretation of BIA-derived data in obese populations, particularly when BIA is used as the sole method for body composition assessment. However, Guedes [50] reported that BIA remains a practical, cost-effective, and noninvasive method for body composition assessment, offering rapid application and good agreement with reference techniques when standardized protocols are followed. Additionally, it allows the estimation of multiple body compartments, requires minimal technical expertise, and is suitable for diverse populations, making it especially useful for routine evaluations and longitudinal monitoring. Despite these practical advantages, the inherent limitations of BIA, particularly its susceptibility to hydration status, lower precision compared to DEXA, and higher margin of error, must be carefully weighed against its accessibility and feasibility in large-scale research and clinical settings. Despite these limitations, this study also presents important strengths. The intervention showed strong ecological validity, as it was conducted in a real-world setting and included a clinically relevant sample with a high prevalence of multimorbidity. Moreover, the program involved fully multidisciplinary and integrated nutritional, psychological, and exercise-related strategies within a single therapeutic framework. Another strength was the use of standardized procedures, the same-trained evaluators in pre- and post-intervention assessments, and excellent measurement reliability, with intraclass correlation coefficients ranging from 0.96 to 0.99. Comprehensive assessments of body composition, physical fitness, and depressive symptoms also strengthen the practical relevance of the findings.

From an applied perspective, the results support the potential usefulness of multidisciplinary lifestyle interventions in primary care and health-promotion settings, particularly for women with obesity, chronic diseases, and functional vulnerability. These findings also reinforce the relevance of resistance training as a core strategy to improve muscle-related and functional outcomes, while psychoeducation appears to be an important adjunct for reducing depressive symptoms and supporting adherence. However, because the exercise program was predominantly resistance-based and included only a limited volume of cardiorespiratory activity, it is plausible that this training specificity explains the absence of significant changes in the physiological responses associated with the 6 min walk test.

Future research should build upon these preliminary findings using randomized controlled trials with larger and more diverse samples, appropriate comparator groups, and extended follow-up periods. Studies designed to disentangle the contribution of each intervention component would also be valuable. In addition, future trials should investigate whether the inclusion of a more structured cardiorespiratory training component enhances physiological adaptation beyond the functional gains observed in walking performance. Taken together, the present findings should be interpreted as preliminary and hypothesis-generating while also providing a relevant basis for the development of more robust confirmatory studies.

## 5. Conclusions

This pre-experimental, single-arm proof-of-concept study aimed to evaluate whether a 12-week multidisciplinary intervention integrating nutritional education, psychoeducation, and resistance training was associated with improvements in health-related physical fitness and depressive symptoms in overweight and obese women aged 45–64 years or older with noncommunicable chronic diseases. Overall, the findings indicate that the intervention was associated with favorable changes in body composition, including increases in lean mass, fat-free mass, and skeletal muscle mass, together with reductions in fat mass, body fat percentage, and visceral fat level. In addition, significant improvements in muscular strength, lower-limb functional performance, flexibility, walking capacity, and depressive symptoms were observed, indicating that the study objectives were achieved.

Moreover, the physiological variables associated with the 6 min walk test did not change significantly, suggesting that the intervention effects were more evident in the morphological, functional, and psychological domains than in acute cardiorespiratory responses. These results are consistent with the exercise characteristics of the program, which was predominantly centered on resistance training and included a limited volume of cardiorespiratory activity. Taken together, these findings support the potential clinical relevance and practical feasibility of an integrated multidisciplinary approach for women with multimorbidity. However, given the exploratory nature of the pre-experimental design and the absence of a control group, the results should be interpreted as preliminary and hypothesis-generating, warranting confirmation in future randomized controlled trials with larger samples and longer follow-up.

## Figures and Tables

**Table 1:** Characteristics of overweight and obese women aged 45–64 years with noncommunicable chronic diseases.

Sample Size	Descriptive—Baseline					
	N	%	Mean	SD	Min.	Max.
Age (years)	31	100	54.7	6.2	45	64
45–49	8	25.8	46.4	1.8	45	49
50–59	14	45.2	54.8	2.5	51	59
60–64	9	29.0	62.1	1.2	60	64
Body Mass (kg)	31	100	84.4	14.7	62.0	122.3
Height (cm)	31	100	160	6	150	176
Overweight (25.0–29.9 kg/m^2^)	7	22.6	27.2	1.6	25.0	29.3
Obese (≥30.0 kg/m^2^)	24	77.4	34.4	4.6	30.1	50.3
Arrhythmia	1	3.2	-	-	-	-
COPD	1	3.2	-	-	-	-
Chronic Venous Insufficiency	3	9.7	-	-	-	-
Depression	10	32.1	-	-	-	-
Diabetes Mellitus	5	16.1	-	-	-	-
Dyslipidemia	12	38.7	-	-	-	-
Hyperuricemia	1	3.2	-	-	-	-
Hypothyroidism	2	6.5	-	-	-	-
Neuropathic Pain	1	3.2	-	-	-	-
Obesity	24	77.4	-	-	-	-
Osteoporosis	1	3.2	-	-	-	-
Systemic Arterial Hypertension	14	45.2	-	-	-	-
Multimorbidity—NCDs	31	100	-	-	2	5
Yes	27	87.1	-	-	2	5
No	4	12.9	-	-	1	1

**Note:** N = absolute frequency; % = relative frequency; SD = standard deviation; Min. = minimum; Max. = maximum; NCDs = noncommunicable chronic diseases; COPD = chronic obstructive pulmonary disease.

**Table 2:** Values of the health-related physical fitness test before and after multidisciplinary intervention in overweight and obese women aged ≥ 45 years with noncommunicable chronic diseases.

Health-Related Physical Fitness Tests	Measurement Times						Δ%
	PRE			POST			
	M	SD	95%CI	M	SD	95%CI	
MIHS (kgf) *	47.9	10.8	44.0–51.9	52.0	9.0	48.7–55.3	11.4
MILTS (kgf) *	55.8	17.8	49.3–62.4	66.3	17.4	59.9–72.7	29.5
CST (rep) *	15.4	3.3	14.1–16.6	20.6	5.3	18.6–22.5	38.7
SR (cm) *	23.9	6.7	21.5–26.4	26.1	9.0	22.8–29.4	9.1
6MWT (m) *	507.2	56.1	486.6–527.8	561.6	49.5	543.5–579.8	11.4

**Note:** Data are expressed as the mean and standard deviation; 95% CI = 95% confidence interval; **Δ%** = delta relative values (pre- minus post-relative values); MIHS = maximal isometric handgrip strength; MILT = maximal isometric lumbar traction strength; CST = Chair Stand Test; SR = sit-and-reach test; 6WMT = six-minute walk test; * = *p* < 0.05 = difference between pre- and post-intervention.

**Table 3:** Physiological Responses in the Six-Minute Walk Test before and after multidisciplinary intervention in overweight and obese women aged 45–64 years with noncommunicable chronic diseases.

Physiological Responses 6MWT	Measurement Times											
	PRE						POST					
	PRE—6MWT			POST—6MWT			PRE—6MWT			POST—6MWT		
	M	SD	95%CI	M	SD	95%CI	M	SD	95%CI	M	SD	95%CI
HR (bpm)	74	10	71–78	121	21	113–128	74	12	69–78	120	14	115–125
SpO_2_ (%)	97	1	97–98	96	2	95–97	97	2	97–98	97	2	96–97
SBP (mmHg)	128	15	123–133	140	21	132–147	133	15	127–138	146	20	139–153
DBP (mmHg)	75	11	71–78	80	10	76–83	76	12	72–81	79	12	74–83

**Note:** Data are expressed as the mean and standard deviation; 95% CI = 95% confidence interval; HR = heart rate; SpO_2_ = peripheral oxygen saturation; SBP = systolic blood pressure; DBP = diastolic blood pressure; PRE = pre-multidisciplinary intervention; POST = post-multidisciplinary intervention; no significant difference between pre- and post-intervention assessments (*p* > 0.05).

## Data Availability

The data will be shared upon request.

## Abbreviations

The following abbreviations are used in this manuscript:

NCDs	Noncommunicable Chronic Diseases
MIHS	Maximal Isometric Handgrip Strength
MILTS	Maximal Isometric Lumbar Traction Strength
CST	Chair Stand Test
SR	Sit-and-Reach Test
6MWT	Six-Minute Walk Test
HR	Heart Rate
SPO_2_	Peripheral Oxygen Saturation
SBP	Systolic Blood Pressure
DBP	Diastolic Blood Pressure
PHQ-9	Patient Health Questionnaire-9
